# Impact of the COVID‐19 pandemic and multiple community lockdowns on total live birth rates and preterm births in Melbourne, Australia

**DOI:** 10.1111/ajo.13527

**Published:** 2022-04-08

**Authors:** Scott Stansfield, Arsheeya Rattan, Ben W. Mol, Daniel L. Rolnik, Atul Malhotra

**Affiliations:** ^1^ Department of Paediatrics Monash University Melbourne Victoria Australia; ^2^ Women's and Newborn Program Monash Health Melbourne Victoria Australia; ^3^ Department of Obstetrics & Gynaecology Monash University Melbourne Victoria Australia

**Keywords:** baby boom, lockdown, prematurity, restrictions

## Abstract

We evaluated the impact of the COVID‐19 pandemic and Melbourne's multiple community lockdowns (between 2020–21) on total live birth rates and preterm births in a large health network. Analysis revealed a decrease in total live birth rates following easing of initial lockdowns, and a sharp increase in births at one stage in between lockdowns. The proportion and number of preterm births (<37 weeks gestation) decreased at the start of initial lockdowns with the strongest decrease after the end of the second lockdown period. Births <34 weeks gestation also decreased during lockdowns, but no significant change was identified for births <28 weeks gestation.

The COVID‐19 pandemic and societal response has led to disruption of all human activity worldwide. It has had an impact on birth rates with most current data showing a decrease in overall birth rates^1^, ^2^ and preterm birth rates^3^, ^4^, ^5^, ^6^ after lockdown measures were introduced in different settings. However, there are also studies which have showed no decrease in preterm birth rates^7^, ^8^, ^9^ and some early research in Australia predicted an increase in overall birth rates in the early months of 2021.^10^ Melbourne has cumulatively had the world's longest lockdown to date (over 260 days) divided over six different periods. In this study, we assessed the impact of the pandemic including the different lockdowns on overall birth rates and proportion of preterm births in a Melbourne health network.

Ethics approval was obtained from Monash Health Human Research Ethics Committee (approval number QA/69113/MonH‐2020‐235157). Live births at Monash Health (Victoria's largest public health network) between 1 January 2018 to 21 October 2021 were included in the analysis. Multiple births were excluded since multiple pregnancies is a strong risk factor for premature births, and if restrictions were associated with a change in the proportion of multiple pregnancies, that may be reflected in the rate of preterm births which would not hold true for singleton births. An interrupted time‐series analysis of weekly live birth rate for all infants and proportion of premature infants <37 weeks was conducted in the R statistical environment (v4.1.2, R Corporation, Vienna, Austria) using an auto‐regressive integrated moving average model (ARIMA) in the forecast package^11^, ^12^ Rates of birth and proportion of premature infants <37 weeks for the four weeks after the starting or end of lockdown periods and the remaining period were compared to birth rate before social distancing measures were first established on 16 March 2020.^13^ The weekly birth rate of premature births at <37, <34 and <28 weeks gestation were also compared over the same time intervals in a separate analysis.

In total, data of 38 525 births were analysed including 35 660 singletons. Multiple births of 790 twins, 12 triplets and one quadruplet birth were excluded. Mean singleton birth rate over the period 1 January 2018 to 21 October 2021 was 190 births/week (95% CI 187–192.8) with seasonal variation. Total birth rates dropped by 15.7 births/week (95% CI 3.9–27.6, *P* = 0.009) in the first four weeks after lockdown one; and 9.3 births/week (95% CI −0.5 to 19.1, *P* = 0.062) in the first four weeks after lockdown two. An increase in total birth rates of 8.1 births/week (95% CI 1.1–15.1, *P* = 0.023) was observed between 1 January 2021 and 20 May 2021 (Fig. 1).

**Figure 1:**
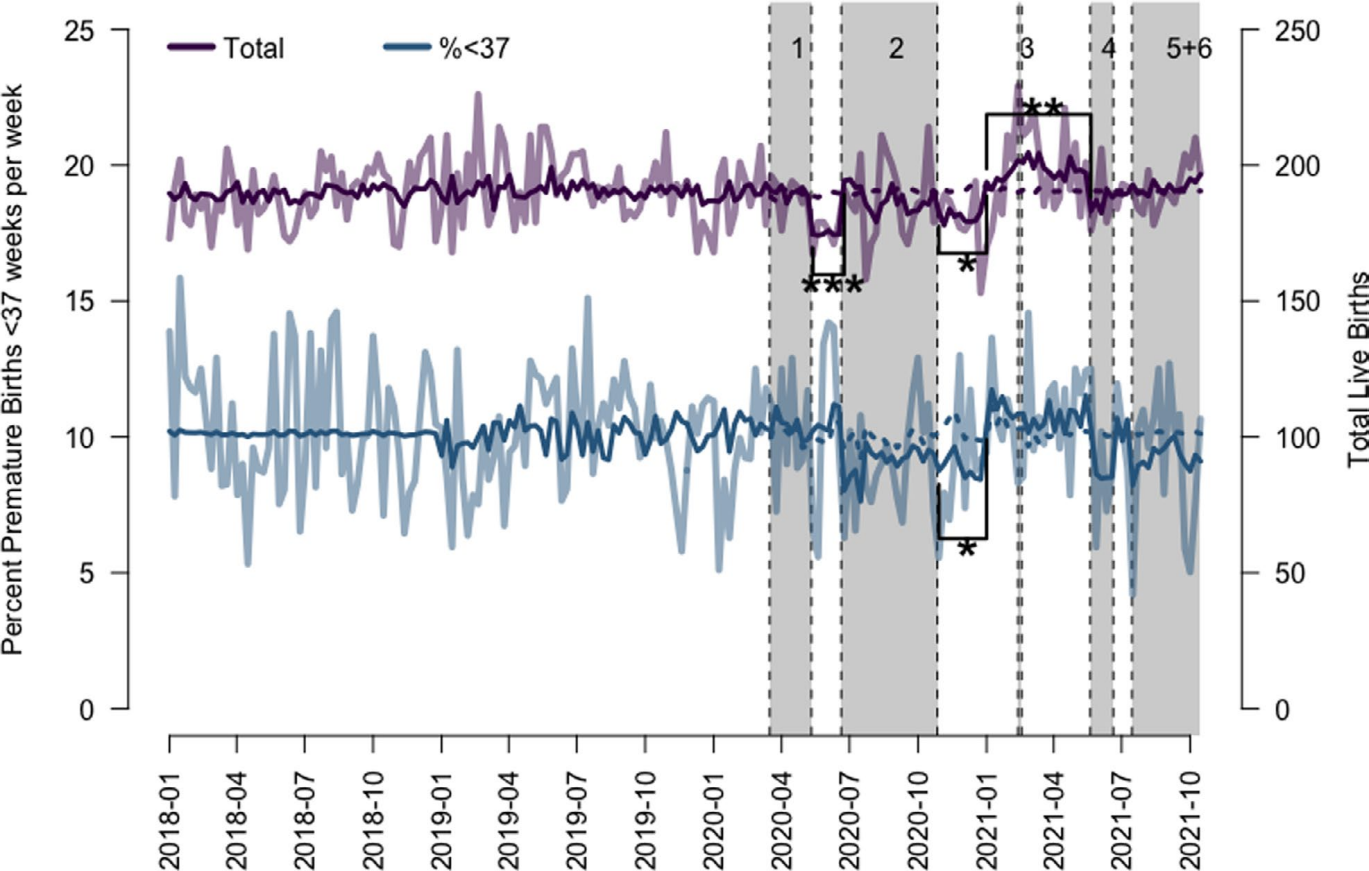
Graph of total live births and proportion of premature births over the pandemic until October 2021. Purple line, solid = interrupted time‐series analysis of all total live births. Purple line, dashed = forecast time‐series analysis of live births prior to lockdown. Blue line, solid = interrupted time‐series analysis of percentage of all premature (<37 weeks completed gestation) birth rate per week. Blue line, dashed = forecast time‐series analysis of percentage of all premature (<37 weeks completed gestation) birth rate per week prior to lockdown. *** denotes *P* < 0.01, ** denotes *P* < 0.05, * denotes *P* < 0.1.

Preterm birth rate <37 weeks was 19.3 births/week (95% CI 18.6–20) accounting for 10.1% (95% CI 9.8–10.4) with seasonal adjustment. Preterm births <37 weeks decreased after lockdown two by 3.8 births/week (95% CI 0.8–6.7, *P* = 0.012) and increased by 1.8 births/week (95% CI 0.24–3.89, *P* = 0.083) between 1 January 2021 and 20 May 2021 (Fig. 2), similarly observed in total births. The proportion of preterm births <37 weeks decreased after lockdown two by 1.4% (95% CI −0.08% to 2.9%, *P* = 0.06). Percentage of preterm births were noted to decrease at the start of lockdowns two (1.4%, *P* = 0.2), four (1.8%, *P* = 0.4) and five (1.4%, *P* = 0.19) but were not statistically significant.

**Figure 2:**
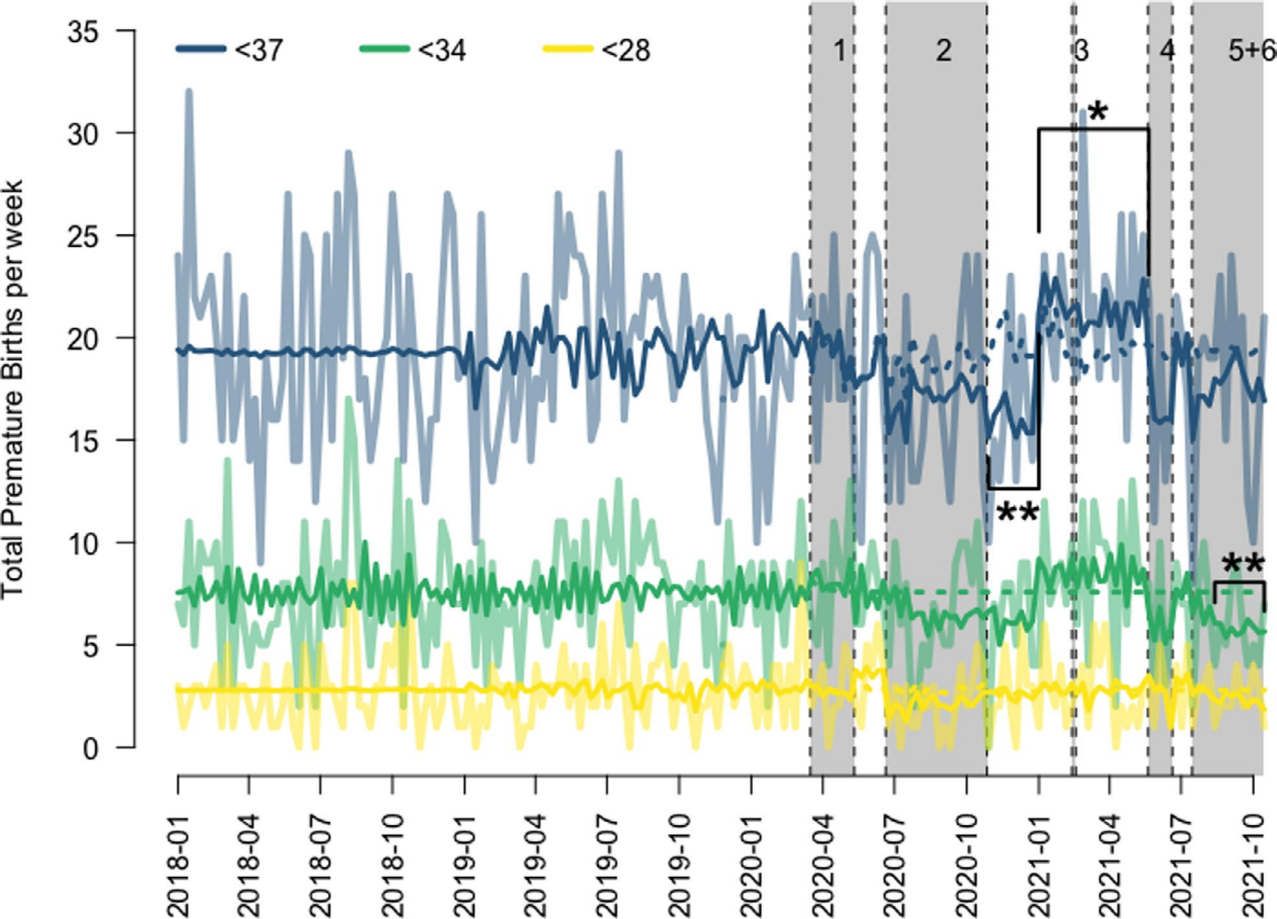
Graph of premature births per week over the pandemic until October 2021. Blue line, solid = interrupted time‐series analysis of premature births <37 weeks. Blue line, dashed = forecast time‐series analysis of premature births <37 weeks prior to lockdown. Green line, solid = interrupted time‐series analysis of premature births <34 weeks. Green line, dashed = forecast time‐series analysis of premature births <34 weeks prior to lockdown. Yellow line, solid = interrupted time‐series analysis of premature births <28 weeks. Yellow line, dashed = forecast time‐series analysis of premature births <28 weeks prior to lockdown. *** denotes *P* < 0.01, ** denotes *P* < 0.05, * denotes *P* < 0.1.

Preterm births <34 weeks accounted for 7.6 births/week (95% CI 7.1–8.1) during the period analysed with no significant seasonal variation. Preterm births were statistically lower by 1.8 births/week (95% CI 0.07–3.6, *P* = 0.042) after four weeks of lockdown five and into lockdown six. Preterm births were noted to be lower during lockdown two (1.2 births/week, *P* = 0.12), after lockdown two (1.4 birth/week, *P* = 0.13) and during lockdown four (first four weeks by 1.6 births/week, *P* = 0.23; remainder 2.4 births/week *P* = 0.35) but were not statistically significant.

Preterm births <28 weeks during the analysed period were 2.8 births/week (95% CI 2.5–3.1) with no significant seasonal variation. Preterm births <28 weeks were not found to be statistically different from the long‐term average during the analysed lockdown periods.

In summary, total live birth rates decreased after initial Melbourne lockdowns; preterm births <37 weeks and <34 weeks decreased after the lockdowns. However, there was no change to the <28 weeks preterm births. These changes could be attributed to multiple factors including economic instability,^14^ decreased access to health care and health‐seeking behaviours^15^ and increased hygiene. Alongside these, the restrictions could have affected health behaviours such as sleep, smoking, alcohol and physical activity^16^ as well as mental health in general^17^ shifting the rates of preterm births. Sexually transmitted infection rates were also impacted by the pandemic,^18^ which could have influenced the rates of preterm births. However, the rate of total live births increased after the easing of lockdown, which might align with initial research predicting an increase in birth rates in the early months of 2021^10^ and the trend for birth rates increasing after other pandemics and epidemics^19^, ^20^ The proportion of preterm birth rates decreased after lockdown two but was not statistically different from seasonal variation during the lockdown. Further work will be needed to identify causes for decreased rates of preterm birth during this period, and how this may inform strategies to decrease preterm birth in the future.
